# Correlates of treatment engagement and client outcomes: results of a randomised controlled trial of nabiximols for the treatment of cannabis use disorder

**DOI:** 10.1186/s13011-022-00493-z

**Published:** 2022-10-08

**Authors:** Llewellyn Mills, Adrian Dunlop, Mark Montebello, Jan Copeland, Raimondo Bruno, Meryem Jefferies, Iain Mcgregor, Nicholas Lintzeris

**Affiliations:** 1 Drug and Alcohol Services, South East Sydney Local Health District, Caringbah, NSW Australia; 2 Specialty of Addiction Medicine, Faculty Medicine, and Health, University of Sydney, Camperdown, NSW Australia; 3 National Drug and Alcohol Research Centre, University of New South Wales, Kensington, NSW Australia; 4 NSW Drug and Alcohol Clinical Research and Improvement Network (DACRIN), NSW Health, St Leonards, Australia; 5 Drug and Alcohol Services, Hunter New England Local Health District, New Lambton, NSW Australia; 6 Priority Research Centre for Brain and Mental Health, School of Medicine and Public Health, University of Newcastle, Callaghan, NSW Australia; 7 Drug and Alcohol Services, Northern Sydney Local Health District, Hornsby, Australia; 8 Mind and Neuroscience - Thompson Institute, University of the Sunshine Coast, Sippy Downs, QLD Australia; 9 University of Tasmania, Hobart, TAS Australia; 10 Drug Health, Western Sydney Local Health District, North Parramatta, NSW Australia; 11 School of Psychology, University Sydney, Camperdown, NSW Australia; 12 Lambert Initiative Cannabinoid Therapeutics, University Sydney, Camperdown, NSW Australia

**Keywords:** Cannabis use disorder, Cannabis dependence, Treatment outcomes, Treatment engagement

## Abstract

**Introduction and aims:**

There is increasing interest and evidence for the use of cannabinoid medications in the treatment of cannabis use disorder, but little examination of the correlates of successful treatment. This paper is a secondary analysis of a randomised placebo-controlled trial of nabiximols for the treatment of cannabis use disorder (CUD), aiming to identify which client and treatment characteristics impact treatment engagement and outcomes.

**Method:**

Bayesian multiple regression models were used to examine the impact of age, gender, duration of regular cannabis use, daily quantity of cannabis, cannabis use problems, self-efficacy for quitting, sleep, mental health, pain measures, and treatment group upon treatment engagement (retention, medication dose, and counselling participation) and treatment outcomes (achieving end-of-study abstinence, and a 50% or greater reduction in cannabis use days) among the 128 clients participating in the 12-week trial.

**Results:**

Among the treatment factors, greater counselling attendance was associated with greater odds of abstinence and ≥ 50% reduction in cannabis use; nabiximols with greater odds of ≥ 50% reduction and attending counselling, and reduced hazard of treatment dropout; and higher dose with lower odds of ≥ 50% reduction. Among the client factors, longer duration of regular use was associated with higher odds of abstinence and 50% reduction, and lower hazard of treatment dropout; greater quantity of cannabis use with reduced hazard of dropout, greater odds of attending counselling, and higher average dose; greater pain at baseline with greater odds of ≥ 50% reduction and higher average dose; and more severe sleep issues with lower odds of ≥ 50% reduction. Males had lower odds of attending counselling.

**Discussions and conclusions:**

These findings suggest that counselling combined with agonist pharmacotherapy may provide the optimal treatment for cannabis use disorder. Younger clients, male clients, and clients with sleep issues could benefit from extra support from treatment services to improve engagement and outcomes.

**Trial registration:**

Australian New Zealand Clinical Trials Registry (ACTRN12616000103460) https://www.anzctr.org.au

**Supplementary Information:**

The online version contains supplementary material available at 10.1186/s13011-022-00493-z.

## Introduction

Cannabis is the world’s most widely used illicit drug with an estimated 4% of the population between 15 and 64 using cannabis at least once in 2019 [1] (12.1% in Australia [2]). A recent meta-analysis of international studies estimated that 22% (95%CI: 18–26%) of people who try cannabis will develop Cannabis Use Disorder (CUD) [3]. In 2016, over 150,000 Australians met criteria for cannabis dependence (equivalent to moderate-severe CUD using DSM5 criteria), at an estimated prevalence of 0.68% (0.6–0.79) of the population [4]. Cannabis Use Disorder is associated with a range of health and social harms [5–8] yet, to date, there are few effective treatment approaches – with over 80% of clients who undertake psychosocial interventions (e.g. cognitive behavioural therapy [CBT], motivational enhancement therapy) [9, 10] or acute withdrawal management [11] returning to regular use within 1–6 months [12–15] of completing treatment. More effective approaches are required for people seeking treatment for cannabis-related problems.

Our research group recently conducted a placebo-controlled randomised trial examining whether clients taking the cannabinoid agonist drug nabiximols for their CUD used less illicit cannabis than clients taking placebo. The results of that trial are reported in detail elsewhere [16, 17], however, we were interested in further examining data from this study to identify which baseline client characteristics were associated with treatment engagement (e.g. medication doses, counselling attendance, treatment retention) and whether client characteristics or treatment conditions (e.g. attendance at counselling) were associated with reductions in cannabis use. Identification of the most important correlates of treatment engagement and cannabis use can assist in better targeting treatment approaches, refining of treatment interventions, or identifying vulnerable client subgroups who require additional support.

Several studies have previously reported the impact of client or treatment characteristics upon client outcomes with psychosocial interventions for cannabis use disorder. Gullo and colleagues demonstrated social-cognitive factors such as client-perceived self-efficacy to quit cannabis were predictive of achieving abstinence [18, 19]. Copeland and Maxwell [20] found that clients with fewer psychological and employment problems, and better family and social supports had better odds of achieving abstinence. In their study of web-based interventions for cannabis users, Jonas and colleagues identified client treatment goals and ‘severity of cannabis use’ (amount used at treatment entry, desire for intoxication) as independently associated with reduced cannabis use during treatment [21].

In recent years there has been a concerted search among researchers for pharmacotherapies that help reduce illicit cannabis use among clients with CUD [22]. A promising direction has been the cannabioind agonist medications, but even these results have been mixed. Dronabinol, a synthetic THC medication, has shown no advantage over placebo in reducing days of cannabis use among individuals with CUD [23, 24], but results for nabiximols, a 1:1 plant-extracted CBD/THC cannabinoid medicine have been more encouraging. In an inpatient randomised controlled trial (RCT; *N* = 51), nabiximols significantly reduced cannabis withdrawal severity and improved odds of withdrawal completion, however most participants had resumed regular cannabis use within 3 months [12], prompting researchers to examine longer term treatment episodes. This was first examined in an early pilot placebo-controlled RCT feasibility study [25]. The study found no significant difference in cannabis use outcomes between the placebo and nabiximols groups, most likely due to lack of power (*N* = 40), however it demonstrated the feasibility of this approach. Most recently our research group conducted a larger RCT of *N* = 128 individuals with CUD. We found that participants receiving nabiximols used illicit cannabis on 18.6 (95%CI: 3.5, 33.7) fewer days during the 84-day, trial, had greater odds of reducing cannabis by 50% or more [16], and that the advantage over placebo was still present 3 months post-treatment [17]. Though promising, replication of these results in additional randomised trials is necessary, and as yet there are still no FDA-approved pharmacotherapies for CUD [22].

Previous research in the area of opioid agonist treatment for opioid use disorder has demonstrated the relationship between higher methadone or buprenorphine doses and reduced unsanctioned opioid use [26]. It is unclear whether similar findings also apply to nabiximols-assisted treatment of cannabis use disorder – with one prior pilot study reporting less cannabis use in clients using higher doses of nabiximols [27].

Multiple systematic reviews have concluded that psychosocial interventions, especially cognitive behavioural therapy (CBT), are effective at reducing cannabis use and severity of dependence among clients with cannabis use disorder [9, 10, 28], and evidence from randomised controlled trials (RCTs) in alcohol, opioid, and stimulant use disorders indicates that counselling (e.g. CBT) in combination with pharmacotherapies is more efficacious than counselling or medication alone [29], however, the impact of counselling in conjunction with pharmacotherapy for cannabis use disorder is yet to be examined.

There is increasing recognition that many people who use cannabis ‘self-medicate’ for a range of concomitant health conditions – most commonly chronic pain, sleep, depression anxiety and stress symptoms [30], and the existence of such underlying conditions for individual clients may impact upon their continued use of cannabis during treatment.

This study is a secondary analysis of a previously reported RCT of nabiximols in the treatment of cannabis use disorder, to examine the extent to which treatment engagement (retention, medication doses, counselling attendance) was associated with client characteristics measured at treatment entry; and the extent to which cannabis-related treatment outcomes (attainment of abstinence and/or marked reductions in cannabis use) are associated with baseline characteristics and/or treatment conditions.

## Methods

Details of study procedures have been described previously [31, 32]. Briefly, cannabis-dependent treatment seekers were randomised to either nabiximols or placebo under double-blind conditions in a 12-week outpatient multisite study, accompanied by psychosocial interventions (structured CBT-based counselling and weekly clinical reviews). The endpoint was the number of self-reported cannabis use days over the 12-week treatment period, collected at 4-weekly research interviews.

### Participants

Participants were treatment seekers who: (i) were 18–65 years old, (ii) met criteria for ICD-10 cannabis dependence [33], (iii) did not meet criteria for another substance use disorder (excluding nicotine or caffeine), (iv) had no severe active medical or psychiatric disorder, and (v) had not received treatment for cannabis use in the previous four weeks [31].

### Materials and measures

#### Demographics and history of cannabis use

Demographic details (age, gender, employment, relationship status, education level) and lifetime history of cannabis use (age first regular use) were collected at baseline research interview.

#### Quantity and frequency of cannabis use

Were assessed using the TimeLine Follow Back (TLFB) [34] technique at 4-weekly research interviews to assess the number of days used in the preceding 28 days, and the average amount of cannabis used on a typical use day (in grams).

#### *The Depression, Anxiety, and Stress Scale (DASS) *[35]

A 21-item questionnaire collected at baseline, consisting of three subscales measuring symptoms of depression, anxiety, and stress, tallied for a total DASS score that represents a “composite measure of negative emotional symptoms” [36]. Higher scores on the DASS indicate more severe symptoms.

#### *The Insomnia Severity Index (ISI) *[37]

Collected at baseline and consisting of 7-items measuring sleep difficulty in the preceding week, with higher scores indicating more severe sleep problems.

#### Short-Form-36 (SF-36) Pain Factor

The Pain Factor of the SF-36 [38] comprises two items, one assessing pain severity and pain interference with usual activities. Higher scores indicate fewer problems, i.e. less pain.

#### *The Cannabis Problems Questionnaire (CPQ) *[39]

The 27-item CPQ measures the physical, psychological, and social consequences of cannabis use on a 0–10 numerical rating scale, with higher scores indicating more severe problems.

#### *The Self-Coping and Efficacy for Quitting Cannabis Questionnaire (QCQ) *[40]

Rates confidence in one’s ability to resist the use of cannabis in a variety of inter-and intra-personal situations, using a 7-point Likert scale. Higher scores indicate greater confidence in the ability to resist cannabis use.

#### Duration of treatment

Was measured weekly (1–12), and refers to participation in study treatment as per protocol.

#### Dose of medication used

Doses of medication (nabiximols or placebo) were prescribed using a flexible client-titrated dosing regimen (up to 32 oromucosal sprays daily, each spray containing 2.7 mg THC and 2.5 mg CBD). Doses used by participants were documented at weekly interviews and the mean dose in Weeks 2–12 was calculated for each participant.

#### Number of counselling sessions

The number of counselling sessions (structured CBT-based individual sessions delivered by trained therapists) attended was recorded for each participant by study staff (maximum of 6 sessions in 12 weeks).

### Statistical analysis

All of the regression models below were conducted using a Bayesian framework.

### Baseline client characteristics

The correlates of treatment engagement and treatment outcome were selected based on client characteristics that have been previously hypothesised to influence cannabis treatment outcomes (see Introduction for summary), with the addition of variables measuring common health conditions for which consumers often reporting self-medicating with cannabis (pain, mental health and sleep conditions), and the minimum number of events per predictor variable (EPV, ‘predictor’ used in the statistical sense rather than implying cause) of 10 recommended for exploratory analyses by Peduzzi and colleagues [41]. The nine covariates used in all analyses were:

1. Duration of Regular Use (at least weekly use; continuous variable measured in decades)
2. Gender (categorical variable; male, female, and non-binary)
3. Treatment group (categorical variable; placebo vs nabiximols)
4. The average amount of cannabis used in the 28 days before baseline (continuous variable in grams per day).
5. Cannabis Problems Questionnaire score (continuous variable; range = 0–270)
6. Self-Coping and Efficacy for Quitting Cannabis Questionnaire score (continuous variable; range = 20–140)
7. SF-36 Pain Score (continuous variable; range = 0–100)
8. DASS total score (continuous variable; range = 0–63)
9. Insomnia Severity Index (continuous variable; range = 0–28)

Covariates whose scales do not have a natural interpretation (CPQ, QCQ, ISI, SF-36, DASS) were converted to *z*-scores. Covariates whose scales *do* have a natural interpretation (e.g. lifetime duration of regular use in years, grams of cannabis used in 28 days before baseline) were left unstandardised for ease of interpretation. All covariates were tested for multicollinearity via a correlation matrix and variance inflation factors. Covariates for each analysis are summarised in eTable 2, supplementary materials.

#### Outcomes

The outcome variables tested in this analysis fell into two broad categories: measures of treatment engagement and measures of outcome.

#### Treatment engagement

Was quantified in three ways:

(i) Duration of treatment. Participants could leave the study on any of the 84 study days, however only the week they left was recorded, making time in treatment a discrete variable representing an underlying continuous variable. When time-to-event data takes this form a discrete-time hazard model with complementary log–log link function is recommended [42]. Discrete-time hazard models are essentially level-means coded logistic regressions with each discrete time period having its own coefficient in the regression model along with any other covariates (in this case the nine covariates mentioned above). If participants remained in the study until Week 12 they were considered to have completed treatment (i.e. censored), hence only covariates for weeks 1–11 were included in the model. Prior distributions for this model were non-informative ‘uniform (-∞, ∞)’ priors on all intercepts (there are no b coefficients in means-level coded models, only intercepts).
(ii) Number of counselling sessions attended. We used aggregated binomial regression to model the influence of the covariates on the number of sessions, a bounded count variable expressing number of sessions attended out of a maximum possible six. To control for exposure − the length of time participants were enrolled in the study and *able* to take part in counselling sessions − the natural logarithm of the number of weeks participants were enrolled in the study was included in the model as an offset variable along with the nine covariates, making ten covariates in total in this analysis. Prior distributions were extremely broad, weakly-regularising ‘t(3, 0, 2.5)’ for the intercept and b’s.
(iii) The average daily dose of study medication. We performed a simple linear regression, regressing the average dose of medication (continuous variable number of sprays per day, 1–32) across weeks 2–12 on the nine covariates. Doses were titrated upwards in Week 1 and hence omitted from the analysis. Prior distributions were extremely broad, weakly-regularising priors: ‘t(3,17.3,10.7)’ for the intercept and b coefficients, and ‘’t(3,0,10.7)’ for the noise distribution.

#### Treatment outcomes

Measures of frequency of cannabis use were the primary end-point for the study. We categorised self-reported number of days of cannabis use in the 28-day period covering Weeks 9–12 (collected at Week-12 interview) into two binary categorical outcomes:

(i) ‘Abstinence’ (no days of cannabis use reported during weeks 9–12 vs > 0 days of cannabis use)
(ii) ‘ ≥ 50% reduction in cannabis use days’ (50% or greater reduction in number of days of cannabis use in weeks compared to days used reported for the 4 weeks prior to the baseline interview vs ≤ 50% reduction).

Missing data were imputed on a ‘worst-case scenario’ basis (a conservative technique often used for imputing missing data in drug treatment trials [43–45]), where participants who dropped out of the study before the final measurement at week 12 were considered as not having achieved the conditions of ‘Abstinent’ or ‘ ≥ 50% reduction in cannabis use days’. Bernoulli logistic regressions were performed, with each of the two binary treatment success variables (abstinence and ≥ 50% reduction) regressed on the nine baseline client characteristics. Number of counselling sessions attended by each participant during the trial and average daily dose of medication were added to the nine baseline characteristics as additional covariates in these regression models. Prior distributions for these Bernoulli models were broad, weakly regularising ‘t(3,0,2.5)’ on the intercepts and ‘uniform(-∞, ∞)’ for the b coefficients.

Bayesian analysis yields no *p*-values, and inferences are made based instead on posterior credibility intervals (similar to confidence intervals). Hence, any coefficients whose 95% credibility intervals (CIs) exclude 0 (in the case of Gaussian regression) or 1 (in the case of binomial regression) will be referred to as ‘noteworthy’ or ‘notable’ rather than ‘significant’. All analyses were performed in R, version 4.0.3, using the base [46], tidyverse [47], survival [48], and brms [49] packages. The brms package is a convenience wrapper for the stan Bayesian statistical software [50], which uses Hamiltonian Monte Carlo sampling methods to generate parameter estimates. Each model parameter was sampled 1000 times (after 1000 warm-up samples) in four chains, yielding 4000 estimates per parameter. Prior distributions for the regression models were the default non-informative priors provided by the brms package, as described above and in eTable 7, supplementary materials. Bayesian model diagnostics − trace plots, R-hat and estimated sample size − were also conducted for each parameter in each model. For interested readers we performed a sensitivity analysis, running equivalent analyses for all the models mentioned above but using classical methods (i.e. Null Hypothesis Significance Testing). Results of these parallel analyses are supplied with supplementary materials (eTables 3-6b) provided with this manuscript along with all data and code.

## Results

### Participants

128 participants were randomised and received at least one dose of trial medication – 67 randomised to placebo, and 61 to nabiximols groups. Sample characteristics have been reported in detail elsewhere [16], but, briefly, at recruitment participants were 35.0 ± 10.9 yrs-old on average (median = 32, IQR: 26,44), 30 (23.4%) were female, and 71 (55.5%) were employed. Participants reported using 2.3 ± 2.1 g of cannabis per day on 25.7 ± 4.5 days in the previous 28 days, with lifetime duration of regular cannabis use of 15.7 ± 9.8 years (median = 14, IQR: 8, 21). There were no notable differences between the nabiximols and placebo groups in any of the variables measured at baseline (see eTable 1, supplementary materials).

Sixty participants (46.9%) completed the 12-week treatment protocol – with similar proportions in the placebo (30/67, 44.8%) and nabiximols (30/61, 49.2%) group. Seventy-seven participants (60.2%) completed the Week-12 research interview, with similar proportions in the placebo (40/67, 59.7%) and nabiximols groups (37/61, 60.7%).

Due to low numbers in the non-binary category, Gender was changed from a three-level categorical (male [*n* = 97], female [*n* = 30], and non-binary [*n* = 1]) to a dichotomous variable (male [*n* = 97; reference group] vs non-male [*n* = 31]) for purposes of analysis.

### Model checks and diagnostics

#### Multicollinearity

The only correlation between covariates that was over *r* = 0.5 was between the DASS Total Score and the Cannabis Problems Questionnaire (*r* = 0.65). However, this correlation was not high enough on its own to indicate multicollinearity problems. Variance inflation factors were < 3 for all covariates in both analyses (VIF range: 1.1–2.0 for both Abstinence and ≥ 50% Reduction criteria) indicating an absence of problematic multicollinearity [51].

#### Bayesian model diagnostics

All models converged well, with stationary well-mixed trace-plots, R-hat < 1.01, and estimated sample size > 1000 for all parameters.

### Correlates of treatment engagement

**Fig. 1:**
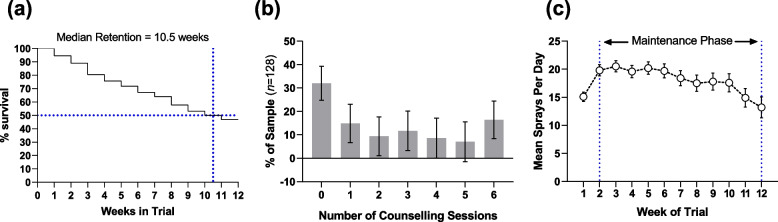
Treatment engagement during the 12-week Trial: (**a**) Treatment Retention, (**b**) number of counselling sessions, (**c**) average dose during weeks 2–12. Note: In (**b**) Error bars represent standard error of a percentage, ${\text{se}}_{\mathrm{\%}}= \sqrt{\frac{\mathrm{p}(1-\mathrm{p}}{\mathrm{n}}} \times 100$, where *p* = proportion of total sample who attended that number of sessions and *n* = count of people who attended that number of sessions. In (**c**) error bars represent standard error

Treatment engagement (duration of treatment, number of counselling sessions, and average medication dose) during the 12-week trial is presented in Fig. 1, and the analysis of correlates of treatment engagement in Table 1.

**Table 1:** Correlates of treatment engagement

	**Outcome**		
**Covariate Name**	**Duration of treatment** HR^a^ (95% CI)	**No. of Counselling Sessions** OR^b^ (95% CI)	**Average Dose** Estimate^c^ (95% CI)
**Duration of Regular Use**, in years	**0.54 (0.41, 0.71)**	1.17 (0.97, 1.40)	0.65 (-1.34, 2.72)
**Gender**, binary, reference group = male (vs non-male^d^)	0.90 (0.45, 1.69)	**1.54 (1.01, 2.36)**	0.44 (-4.15, 5.02)
**Treatment group**, binary; reference group = Placebo (vs Nabiximols)	**0.60 (0.37, 0.96)**	**1.41 (1.02, 1.97)**	-0.48 (-4.15, 3.22)
**Average quantity of cannabis used**, in grams per day	**0.83 (0.70, 0.95)**	**1.11 (1.01, 1.21)**	**1.05 (0.16, 1.98)**
**Cannabis Problems Questionnaire**, as *z*-score	0.99 (0.70, 1.42)	0.90 (0.72, 1.13)	-0.67 (-2.98, 1.80)
**Self-coping and efficacy for quitting questionnaire**, as *z*-score	0.96 (0.73, 1.26)	0.98 (0.83, 1.16)	-0.78 (-2.54, 1.06)
**SF-36 Pain score**, as *z*-score	1.21 (0.95, 1.56)	0.93 (0.78, 1.12)	**-2.89 (-4.74, -0.99)**
**DASS total score**, as *z*-score	1.10 (0.77, 1.55)	1.06 (0.83, 1.34)	-0.78 (-3.31, 1.74)
**Insomnia Severity Index**, as *z*-score	1.07 (0.83, 1.40)	0.96 (0.79, 1.17)	0.56 (-1.56, 2.67)

^a^z-score = (raw score – mean)/sd for variable in question. Hazard Ratio: difference in rate of dropout from study given 1-unit change in covariate (when noteworthy 95% CI excludes 1). Coefficients for each of the 11 time periods (weeks 1–11) are not included in this table (see eTable 3 Supplementary Materials)

^b^Odds Ratio: difference in the odds or attending an extra counselling session, given a 1-unit change in the covariate (when noteworthy, 95% CI excludes 1)

^c^change in estimated mean sprays per day for 1-unit change in covariate (when noteworthy 95% CI excludes 0)

^d^non-male = female (*n* = 30) + non-binary (*n* = 1), collapsed together due to low numbers in non-binary group

#### Duration of treatment

The median treatment duration in the study was 10.5 weeks. The CIs for all 11 time period coefficients (not included in Table 1) excluded 1, indicating a non-zero hazard of treatment dropout. Coefficients and CIs for these are included in eTable 3, supplementary materials. A longer history of regular cannabis use (Hazard Ratio [HR] = 0.54, 95%CI: 0.41, 0.71)*,* being randomised to receive nabiximols (HR = 0.60, CI: 0.37, 0.96) and using a greater quantity of cannabis on days when cannabis was used (HR = 0.83, CI: 0.70, 0.95) were all associated with notably reduced hazard of treatment dropout.

#### Number of counselling sessions attended

Participants took part in 2.4 ± 2.2 counselling sessions on average. Female or non-binary clients (Odds Ratio [OR] = 1.54, CI: 1.01, 2.36), clients receiving nabiximols (OR = 1.41, CI: 1.02, 1.97), and clients who used a greater quantity of cannabis on days when they used cannabis (OR = 1.11, CI: 1.01, 1.21) all had greater odds of attending any given counselling session.

#### Average dose of study medication

The mean dose across the 12-week trial across all participants was 18.1 ± 9.5 sprays per day (Placebo = 18.9 ± 9.3; Nabiximols = 18.2 ± 9.5, a non-noteworthy estimated difference of 0.93 sprays per day [CI: -4.41, 2.47]). Greater quantity of cannabis on cannabis use days (estimate = 1.05; CI: 0.16, 1.98) and more severe pain at baseline (estimate = 2.89; CI: -4.74, -0.99) were both associated with notably more sprays of medication per day on average.

### Correlates of treatment outcomes

During weeks 9–12, abstinence was achieved by 17 (13.3%) of the 128 participants and ≥ 50% Reduction by 31 participants (24%). The results of the regression models for the two treatment outcomes – abstinence during weeks 9–12 and ≥ 50% reduction in days’ used relative to baseline – are presented in Table 2.

**Table 2:** Correlates of client outcomes regarding cannabis use (Abstinence and ≥ 50% Reduction^a^)

**Variable**	**Abstinent** (*n* = 17)	**Non-abstinent** (*n* = 111)	**OR**^b^ **(95% CI)**	** ≥ 50% reduction** (*n* = 31)	** < 50% reduction** (*n* = 97)	**OR**^b^ **95% CI**
**Duration Regular Use**, in decades, M (SD)	2.22 (1.12)	1.46 (0.87)	**3.03 (1.36, 7.29)**	1.84 (1.05)	1.47 (0.89)	**2.87 (1.39, 6.60)**
**Gender**, *n* (%)						
Female	9 (30.00)	21 (70.00)	4.12^c^ (0.75, 24.30)	12 (40.00)	18 (60.00)	2.10^c^ (0.49, 9.46)
Male	8 (8.25)	89 (91.75)		19 (19.59)	78 (80.41)	
Non-binary	0 (0.00)	1 (100.00)		0 (0.00)	1 (100.00)	
**Treatment Group**^d^, *n* (%)						
Nabiximols	10 (16.39)	51 (83.61)	2.27 (0.50, 11.51)	20 (32.79)	41 (67.21)	**4.06 (1.21, 14.95)**
Placebo	7 (10.45)	60 (89.55)		11 (16.42)	56 (83.58)	
**Average quantity of cannabis used**, gms, M (SD)	1.89 (1.54)	2.41 (2.13)	0.91 (0.53, 1.42)	1.66 (1.44)	2.56 (2.19)	0.75 (0.46, 1.12)
**Cannabis Problems Questionnaire**, z, M (SD)	0.03 (1.13)	-0.00 (0.98)	0.72 (0.26, 1.97)	-0.04 (1.00)	0.01 (1.01)	0.96 (0.44, 2.09)
**Self-Coping and Efficacy for Quitting**, z, M (SD)	0.29 (1.29)	-0.04 (0.95)	1.50 (0.75, 3.15)	0.03 (1.18)	-0.01 (0.95)	0.85 (0.47, 1.53)
**SF-36 Pain score**, z, M (SD)	-0.55 (1.03)	0.08 (0.97)	0.41 (0.16, 1–01)	-0.22 (1.01)	0.07 (0.99)	**0.40 (0.18, 0.83)**
**DASS Total Score**, z, M (SD)	0.27 (1.03)	-0.04 (0.99)	0.98 (0.34, 2.79)	0.04 (1.08)	-0.01 (0.98)	0.65 (0.27, 1.50)
**Insomnia Severity Index**, z, M (SD)	0.19 (1.11)	-0.03 (0.98)	0.68 (0.26, 1.69)	-0.11 (1.09)	0.03 (0.97)	**0.39 (0.18, 0.82)**
**Rate of Counselling Attendance**, average number of sessions per fortnight (14 days), M (SD)	0.23 (0.22)	0.11 (0.27)	**5.31 (1.45, 22.19)**	0.18 (0.11)	0.11 (0.14)	**3.82 (1.30, 12.15)**
**Average Dose weeks 2–12**, sprays per day, M (SD)	14.58 (8.08)	18.70 (9.60)	0.92 (0.83, 1.01)	14.68 (8.95)	19.29 (9.41)	**0.91 (0.84, 0.98)**

^a^z-score = (raw score – mean)/sd for variable in question. For both the abstinence criteria and ≥ 50% reduction criteria there were two potential ways of meeting criteria for non-success: (i) using illicit cannabis at least once in the previous 28 days (ii) dropping out of the study early; that is, failing to complete the week 12 research interview

^b^*OR* Odds ratio. Each coefficient represents the increase in odds of either abstinence during weeks 9-12or ≥ 50% reduction associated with a 1-unit increase in the covariate

^c^for analysis the gender variable was collapsed from a three-level categorical into a two-level categorical: male (ref) vs non-male (female *n* = 30 + non-binary *n* = 1)

^d^placebo (ref) vs nabiximols

Clients with a longer history of regular cannabis use and a greater rate of counselling had notably greater odds of achieving both abstinence (Duration of reg. use: OR = 3.03, CI: 1.36, 7.29; Rate of couns. attendance: OR = 2.87, CI: 1.39, 6.60) and/or a ≥ 50% reduction in days used (Duration of reg. use: OR = 5.31, CI: 1.45, 22.19; Rate of couns. attendance: OR = 3.82, CI: 1.30, 12.15).

Clients who received nabiximols (OR = 4.06, CI: 1.21, 14.95), had more severe pain at baseline (OR = 0.40, CI: 0.18, 0.83), less severe sleep problems (OR = 0.39, CI: 0.18, 0.82), and who used less study medication (OR = 0.91, CI: 0.84, 0.98) all had notably greater odds of reducing their cannabis use frequency by 50% or more (but not of achieving abstinence).

## Discussion

This secondary analysis of data from an RCT of nabiximols for the treatment of cannabis use disorder provides some insights into how treatment might be tailored to individual client characteristics and identifies client and treatment characteristics associated with clinically meaningful reductions in cannabis use. A summary of the results is presented in Table 3.

**Table 3:**
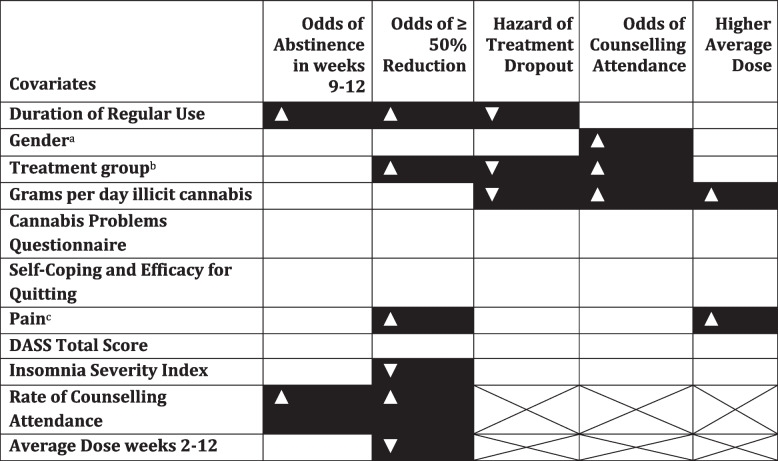
Summary of noteworthy correlates of treatment engagement and outcomes

Black boxes indicate covariates whose 95% credibility interval excluded 1 (or 0 in the case of Average Dose).▼indicates a negative association with the outcome variable in question and the covariate,▲indicates a positive association. X’d out cells indicate that the covariate in question was not included in the model

^a^male gender (*n* = 97/128) vs not male (female = 30/128 + non-binary = 1/128) with male = 0 and non-male = 1

^b^placebo (*n* = 67) vs nabiximols (*n* = 61) with the placebo group = 0 and nabiximols = 1

^c^Higher SF-36 scores indicate better outcome, in this case less pain. This can be confusing, therefore, to save confusion we have reversed the direction of the SF-36 so that the arrow represents the change in odds of reducing cannabis use associated with higher pain at baseline. So, more pain at baseline was associated with greater odds of reducing cannabis and greater average dose of medication

Let us first turn our attention to the client factors. Clients with a longer lifetime duration of regular cannabis use were more likely to stay in treatment and reduce their illicit cannabis use by a clinically meaningful amount. Age and duration of regular use were highly correlated in our sample (*r* = 0.80), and if we consider duration of regular use to be a proxy for age, our finding is consistent with past research into opioid, cocaine, methamphetamine, and alcohol treatment showing that older clients tend to stay in treatment longer [52–55].

Evidence suggests that the life experiences that come with maturity − finding a long-term partner, having children, securing long-term employment – create a stronger motivation for people to reduce their alcohol and drug use [56, 57]. The majority of clients who sought treatment in our study were over 30 years old, started using regularly in their teens, and had been using cannabis regularly for over a decade. It may be that the older among them had reached the point in their lives and cannabis use ‘careers’ where they were ready to engage with treatment and reduce their use.

Clients who used a greater quantity of cannabis each day prior to enrollment tended to remain in treatment longer, attend more counselling, and use a higher dose of medication. This is an encouraging finding as it suggests that those clients with more severe dependence engaged in more intensive treatment.

The women in our study participated in more counselling on average than the men. This is consistent with findings from mental health research showing that men are more reluctant to seek psychological treatment than women [58].

There are frequent reports in the literature of cannabis use as a form of self-medication for chronic pain conditions [59]. In our study those reporting greater pain at baseline used less illicit cannabis and more medication during the 12-week period, suggesting the possibility that these clients substituted illicit cannabis with medication to address their pain symptoms. Given that nabiximols is a buccal spray which, like most forms of prescribed cannabis, has a more favourable safety profile than inhaled routes of administration and keeps clients in regular contact with health professionals, this substitution effect is an encouraging finding.

Interestingly, unlike pain, clients with more severe sleep problems were *less* likely to reduce their cannabis use – reinforcing findings from previous studies indicating that sleep problems are associated with poorer long-term outcomes in treatment for cannabis and other substance use disorders [60–62].

Now we turn to the treatment factors. There was an association in our study between attendance at counselling and reduced cannabis use, and previous research identifies attendance at counselling as a positive prognostic factor [9, 10, 28]. Whilst also an encouraging finding, the causal direction of this association is unclear. It may be that counselling provided participants with greater skills and motivation to reduce their cannabis use; or that those who reduced their cannabis use were more willing to engage in treatment (experiencing perceived benefits of treatment); or alternatively that engagement with counselling may be an index of motivation to engage with treatment and reduce cannabis use, reflecting a third, unmeasured motivational factor [63]. Whatever the direction of causation, our finding suggests that counselling should continue to be encouraged in clients seeking treatment for cannabis use disorder [63].

We have previously reported the finding that nabiximols is associated with reduced cannabis use relative to placebo [16], however this is the first study to find an association between a cannabinoid medication and increased treatment engagement: staying in treatment longer and attending more counselling sessions. This echoes findings demonstrating that opioid agonists increase retention in treatment for opioid use disorder [64].

Contrary to the findings of a previous pilot RCT [27], in our study clients who used higher doses of medication (nabiximols or placebo) had lower odds of reducing frequency of use by 50% or more. However, as our study used a flexible dosing regimen, it was not designed to address the issue of dose response, which requires ‘fixed dose’ conditions with comparisons between randomly allocated groups.

Somewhat surprisingly, client-rated severity of cannabis-related problems, depression, anxiety and stress symptoms, and self-efficacy regarding the ability to quit were not independently related to treatment outcomes, contrary to previous studies of psychosocial interventions [18–21]. It may be that, at baseline, variation within these constructs was not sufficient in our sample to have meaningful associations with later behaviour, or that nabiximols may have reduced the influence of these constructs on outcomes, or possibly that previous studies have overstated the influence of these constructs on outcomes. Further research is necessary to determine the conditions under which these constructs are correlated with successful treatment outcomes and engagement.

What are the implications of these findings for clinical practice?

The fact that both counselling attendance and nabiximols were independently associated with reduced illicit cannabis use and longer stay in treatment, along with the finding that nabiximols was associated with increased counselling attendance, suggests that combining nabiximols with counselling may deliver better results when treating cannabis use disorder than either approach alone. This matches findings from a meta-analysis showing that combined pharmacotherapy and counselling was the most efficacious treatment across a range of substance use disorders [29]. As for the client factors, our findings suggest that extra attention should be made to engage with and retain in treatment clients with poorer outcomes: those who are younger, who are male, and who have pre-existing sleep issues.

### Limitations

Our study has limitations. There were high rates of attrition from research interviews such that only 60% of participants completed the Week 12 interview. We imputed missing data at Week 12 interview using a ‘worst-case scenario’, in which any participant who did not complete this interview was assigned as not meeting the criteria for treatment success. Fortunately, there were similar rates of follow-up between active and placebo groups, such that imputed data were evenly distributed against the two groups. Nevertheless, further studies are required to replicate these findings. Another limitation is the reliance on self-report for the primary endpoint of days of cannabis use. The use of nabiximols (a medication containing THC) prevents the use of THC or its metabolites to corroborate self-report cannabis use. Nevertheless, the validation of self-reported cannabis use in the placebo arm has been reported previously [32]. A further limitation is that this secondary analysis of data was not pre-registered. However, while pre-registration does create transparency in the research process and safeguard against researcher bias, the absence of pre-registration on its own does not indicate the presence of these biases. Finally we did not measure some potential correlates of successful treatment identified in prior literature, such as degree of social support, stability of interpersonal relationships, or employment. Future studies examining correlates of treatment engagement and outcomes should examine the influence of these factors in addition to the correlates we measured.

## Conclusions

Our findings suggest that combining cannabinoid medication with psychosocial interventions may be the optimal treatment approach for cannabis use disorder, with each approach independently associated with reduced cannabis use during treatment. It seems as if younger clients, male clients, and clients with sleep issues may benefit from extra support from services to improve their treatment engagement and outcomes. More research is required to better understand the way that clients with cannabis use disorder and chronic pain use their cannabinoid medication.

## Supplementary Information

**Additional file 1.**

**Additional file 2.**

## Data Availability

The datasets supporting the conclusions of this article are included within the article and the additional files.

## Abbreviations

CBD: Cannabidiol
CBT: Cognitive Behavioural Therapy
CPQ: Cannabis Problems Questionnaire
DASS: Depression Anxiety Stress Scale
EPV: Events Per Predictor index
ISI: Insomnia Severity Index
SF-36: Short Form 36
QCQ: Sell-coping and Efficacy for Quitting Cannabis Questionnaire
RCT: Randomised controlled trial
TLFB: Timeline follow-back
THC: Tetrahydrocannabinol
