# Supplementary material for: Correlates of treatment engagement and client outcomes: results of a randomised controlled trial of nabiximols for the treatment of cannabis use disorder
Source: Subst Abuse Treat Prev Policy. 2022 Oct 8;17:67. doi: 10.1186/s13011-022-00493-z (PMC9548192; doi:10.1186/s13011-022-00493-z)
Supplement: Supplementary file 1 — Additional file 1. [file 13011_2022_493_MOESM1_ESM.docx]

**Correlates of treatment engagement and client outcomes: Results of a randomised controlled trial of nabiximols for the treatment of cannabis use disorder**

**Supplementary Materials**

Authors note: The following supplementary materials document includes

1. eTable 1: Shows baseline characteristics in the two groups, placebo and nabiximols, and average across groups.
2. eTable 2: Shows which covariates were included in which analysis
3. eTables 3-6b: These tables constitute a sensitivity analysis, comparing the results from the analysis presented in the main paper to the results of equivalent NHST analysis. These comparator analyses were conducted using the brm() function in the R package brms, using the default wide, weakly-regularising priors prescribed by the package. The reader will notice that there is no difference in the overall pattern of results – which covariates had noteworthy associations with the outcome in question, here defined as the 95% credibility interval excluding 0 (for Gaussian regression (e.g. dose of medication) or 1 for the remaining outcomes– between the Bayesian and classical approaches.
4. eTable 7: Priors used for each of the Bayesian regression models.

**eTable 1**: Demographic and Baseline Characteristics. Numbers for each group and across entire sample are as indicated for the demographic questionnaire unless otherwise indicated.

| ***Demographic*** |  |  |  |  |  |  |
| --- | --- | --- | --- | --- | --- | --- |
|  | Nabiximols (*n*=61) | Placebo  (*n*=67) | Total  (*n*=128) | *p^a^* | *BF^b^* | *BF_null_^c^* |
| **Age**, mean (SD), y (range: 19 – 66) | 36.22 (11.54) | 33.84 (10.32) | 34.96 (10.93) | 0.23 | 0.38 | 2.66 |
| **Gender**, n (%)  Female  Male  Other | 16 (26.2)  45 (73.8)  0 (0.0) | 14 (20.9)  53 (77.6)  1 (1.5) | 30 (23.4)  97 (75.8)  1 (0.8) | 0.51 | 0.03 | 38.03 |
| **Country of Birth**, n (%)  Australia  Other | 51 (83.6)  10 (16.4) | 56 (83.6)  11 (16.4) | 107 (83.6)  21 (16.4) | 1.0 | 0.16 | 6.15 |
| **Aboriginal/Torres Strait Islander**, n (%)  Yes  No | 4 (6.6)  57 (93.4) | 6 (9.0)  61 (91.0) | 10 (7.8)  118 (92.2) | 0.86 | <0.01 | 1430.02^c^ |
| **Highest level of Education**, n (%)  Primary  Secondary/Yr10  Secondary/Yr12  Tertiary | 2 (3.3)  20 (32.8)  12 (19.7)  27 (44.3) | 4 (6.0)  20 (29.9)  21 (31.3)  22 (32.8) | 6 (4.7)  40 (31.2)  33 (25.8)  49 (38.3) | 0.25 | 0.03 | 33.02 |
| **Main Source of Income**, n (%)  Full-Time Employment  Part-Time Employment  Benefits  Pension  Other | 19 (31.1)  16 (26.2)  12 (19.7)  7 (11.5)  7 (11.5) | 26 (38.8)  10 (14.9)  16 (23.9)  10 (14.9)  5 (7.5) | 45 (35.2)  26 (20.3)  28 (21.9)  17 (13.3)  12 (9.4) | 0.81 | 0.23 | 4.24 |
| **Marital Status**, n (%)  Single  DeFacto  Married  Separated  Divorced | 34 (55.7)  13 (21.3)  5 (8.2)  7 (11.5)  2 (3.3) | 39 (58.2)  16 (23.9)  11 (16.4)  1 (1.5)  0 (0.0) | 73 (57.0)  29 (22.7)  16 (12.5)  8 (6.2)  2 (1.6) | 0.27 | 0.46 | 2.15 |
| **Living Situation**, n (%)  Renting  Own House/Mortgage  Homeless | 31 (50.8)  14 (23.0)  16 (26.2) | 38 (56.7)  13 (19.4)  16 (23.9) | 69 (53.9)  27 (21.1)  32 (25.0) | 0.79 | <0.01 | 143.45 |
| **Children**, n (%)  Yes  No | 21 (34.4)  40 (65.6) | 23 (34.3)  44 (65.7) | 44 (34.4)  84 (65.6) | 1.0 | 0.21 | 4.82 |
| **Legal Situation**, n (%)  No legal problems  Awaiting Trial/Sentencing  Other | 59 (96.7)  0 (0.0)  2 (3.3) | 61 (91.0)  4 (6.0)  2 (3.0) | 120 (93.8)  4 (3.1)  4 (3.1) | 0.33 | 0.24 | 4.17 |
| ***Baseline Cannabis Use Variables*** |  |  |  |  |  |  |
|  |  |  |  | *p^a^* | *BF^b^* | *BF_null_^c^* |
| **Number of days used in last 28**, mean (SD), days/28 | 25.85 (4.58) | 25.63 (4.53) | 25.73 (4.54) | 0.78 | 0.19 | 5.11 |
| **Age of first regular use**, mean (SD) years-old | 20.23 (7.31) | 18.42 (5.50) | 19.25 (6.46) | 0.13 | 0.58 | 1.71 |
| **Duration of regular use**, mean (SD), years | 15.60 (8.85) | 14.60 (9.77) | 15.10 (9.27) | 0.58 | 0.23 | 4.40 |
| **Average grams per day**, mean (SD), g | 2.04 (1.36) | 2.60 (2.53) | 2.34 (2.07) | 0.12 | 0.55 | 1.81 |
| **Cannabis Withdrawal Scale**, mean (SD), 19q; max=190 | 64.75 (43.52) | 65.13 (36.98) | 64.95 (40.06) | 0.64 |  |  |
| **Marijuana Craving Questionnaire**, mean (SD), 12q; max=72) | 34.69 (17.86) | 36.15 (16.42) | 35.45 (17.07) | 0.63 | 0.21 | 4.76 |
| **Cannabis Problems Questionnaire**, mean (SD), 27q; max=270) | 111.95 (52.77) | 117.84 (44.65) | 115.04 (48.58) | 0.50 | 0.23 | 4.28 |
| **Coping and Efficacy for Quitting**, mean (SD), 20q; max = 120) | 43.75 (23.92) | 43.52 (22.05) | 43.63 (22.87) | 0.96 | 0.19 | 5.28 |
| **Route of Administration**, n (%)  Joint  Bong  Vaporizer  Pipe  Eating  Other | 16 (26.2)  43 (70.5)  1 (1.6)  1 (1.6)  0 (0.0)  0 (0.0) | 14 (20.9)  51 (76.1)  1 (1.5)  0 (0.0)  1 (1.5)  0 (0.0) | 30 (23.4)  94 (73.4)  2 (1.56)  1 (0.8)  1 (0.8)  0 (0.0) | 0.64 | <0.01 | >1000 |
| ***Other Variables*** |  |  |  | *p^a^* | *BF^b^* | *BF_null_^c^* |
| **Nicotine dependence**, n (%), (Fagerstrom)  Low  Low-Moderate  Moderate  High | 31 (50.82)  11 (18.03)  15 (24.59)  4 (6.56) | 44 (65.67)  10 (14.93)  12 (17.91)  1 (1.49) | 75 (58.59)  21 (16.41)  27 (21.09)  5 (3.91) | 0.24 | 0.10 | 10.40 |
| **Alcohol dependence**, n (%), (AUDIT)  Low risk  Risky  High Risk  Very High Risk | 46 (75.41)  14 (22.95)  1 (1.64)  0 (0.00) | 54 (80.60)  9 (13.43)  3 (4.48)  1 (1.49) | 100 (78.13)  23 (17.97)  4 (3.13)  1 (0.78) | 0.33 | 0.01 | 71.81 |
| **Insomnia Severity Index**, n (%)  No Insomnia  Subthreshold Insomnia  Moderate Insomnia  Severe Insomnia | 16 (26.23)  13 (21.31)  24 (39.34)  8 (13.11) | 18 (26.87)  19 (28.36)  21 (31.34)  9 (13.43) | 34 (26.56)  32 (25.00)  45 (35.16)  17 (13.28) | 0.75 | 0.05 | 21.01 |
| **DASS**, mean (SD), average item score; max=3  Depression  Anxiety  Stress | 1.26 (0.84)  1.13 (0.72)  1.70 (0.84) | 1.15 (0.82)  1.14 (0.66)  1.70 (0.80) | 1.20 (0.83)  1.13 (0.69)  1.72 (0.81) | 0.44  0.92  0.68 | 0.24  0.19  0.20 | 4.03  5.19  4.90 |
|  |  |  |  |  |  |  |
| **Sheehan Disability Scale**, mean (SD), 3q; max=30 | 12.82 (7.37) | 14.12 (8.18) | 13.50 (7.81) | 0.35 | 0.28 | 3.54 |
| **SF-36**, mean (SD), average item score; max=100  Physical Functioning  Role Limitations Due to Physical Health  Role Limitations Due to Emotional Problems  Energy/fatigue  Emotional Well-being  Social functioning  Pain  General Health | 86.15 (20.72)  34.43 (41.37)  48.09 (45.75)  40.82 (19.26)  54.56 (18.13)  58.81 (26.91)  70.45 (25.64)  53.85 (21.59) | 87.22 (18.41)  36.57 (42.27)  48.26 (44.68)  43.43 (18.34)  55.52 (20.33)  54.59 (30.46)  69.81 (26.17)  49.33 (19.65) | 86.71 (19.47)  35.57 (41.69)  48.18 (45.01)  42.19 (18.76)  55.06 (19.24)  56.60 (28.79)  70.12 (25.82)  51.48 (20.64) | 0.76  0.77  0.98  0.43  0.78  0.41  0.89  0.22 | 0.20  0.20  0.19  0.25  0.20  0.26  0.19  0.38 | 5.07  5.09  5.29  4.00  5.10  3.88  5.24  2.63 |
| **Brief Pain Inventory** |  |  |  |  |  |  |
| Pain other than everyday pain in last week?  Yes  No | 31 (50.8)  30 (49.2) | 37 (55.2)  30 (44.8) | 68 (53.1)  60 (46.9) | 0.75 | 0.25 | 4.06 |
|  |  |  |  |  |  |  |
| Pain severity, mean (SD), max=10; Valid=68; Nabiximols=31, Placebo=37  Pain relief, mean (SD), as %; Valid=68; Nabiximols=31, Placebo=37  Pain Interference, mean (SD), max=10; Valid=64; Nabiximols=30, Placebo=34 | 4.15 (1.40)  40.80 (36.05)  3.98 (2.81) | 4.00 (1.54)  36.79 (38.30)  4.16 (2.66) | 4.07 (1.47)  38.68 (36.95)  4.08 (2.71) | 0.68  0.70  0.80 | 0.27  0.29  0.26 | 3.73  3.39  3.80 |
| **PTSD Screen**, mean (SD), 4q; max=4 | 1.46 (1.56) | 1.62 (1.66) | 1.54 (1.60) | 0.59 | 0.21 | 4.59 |
| **OTI Crime** |  |  |  |  |  |  |
| Drug-related crime in last month, n (%)  Yes  No | 21 (34.43)  40 (65.57) | 24 (35.82)  43 (64.18) | 45 (35.16)  83 (64.84) | 1.0 | 0.21 | 4.73 |
| Non-Drug crime in the last month, n (%), Valid=127, Nabiximols=61, Placebo=66  Yes  No | 60 (98.36) | 3 (4.55)  63 (95.45) | 123 (96.85) | 0.67 | 0.11 | 8.76 |
| Number of convictions, mean (SD), Valid=55; Nabiximols=26, Placebo=29 | 2.27 (4.81) | 2.97 (5.63) | 2.64 (5.23) | 0.62 | 0.30 | 3.32 |
| **Absenteeism^d^**, mean (SD), Valid=81; Nabiximols=40, Placebo=41  Absolute  Relative | 16.12 (33.35)  0.20 (0.37) | 9.51 (44.66)  0.07 (0.47) | 12.78 (39.38)  0.13 (0.42) | 0.45  0.18 | 0.30  0.51 | 3.38  1.96 |

***a:*** *p-values derived from chi-square tests for categorical outcomes and t-tests for continuous outcomes.* ***b:*** *BF = Bayes Factor: alternative hypothesis/null hypothesis. Odds ratio of how much more likely the alternative hypothesis (of any difference) is than the null hypothesis (of no difference) given the data. Values ≥3 constitute substantial evidence in favour of the alterative hypothesis (Kass & Raftery, 1995), values < 3 are inconclusive.* ***c:*** *BF_null_ = Bayes Factor: null hypothesis/alternative hypothesis. Odds ratio of how much more likely the null hypothesis (of no difference) is than the alternative hypothesis. Values ≥3 constitute substantial evidence in favour of the null hypothesis, values < 3 are inconclusive.* ***d:*** *Absolute absenteeism = expected hours worked in last 28 days – actual hours worked in last 28 days (positive value indicates number of hours expected > actual). Relative absenteeism = absolute absenteeism/expected hours in last 28.*

**Interpretation:** For most variables there was substantial evidence (i.e. Bayes Factor ≥ 3.0) of there being no difference between the placebo and the nabiximols groups. The only variables where the *BF_null_* fell below < 3.0 were Relative Absenteeism, Age of first regular use, Average grams per day, Marital Status, and Age. However, it is important to note that in all these cases the Bayes Factor for the alternative hypothesis did not meet criteria for noteworthiness (i.e. where *BF_alternative_* was not ≥ 3.0) and the *p*-values were not <.05, meaning that results for this test were merely inconclusive, *not* that there was a significant difference between the groups for these variables

**eTable 2:** Covariates included in each analysis

|  | **Outcome variable** | | | | |
| --- | --- | --- | --- | --- | --- |
|  | **Treatment**  **Engagement** | | | **Treatment**  **Outcome** | |
| **Covariate** | Treatment retention | No. of Counselling Sessions | Dose | Abstinence | ≥50% Reduction |
| Duration of Regular Use | 🗸 | 🗸 | 🗸 | 🗸 | 🗸 |
| Gender | 🗸 | 🗸 | 🗸 | 🗸 | 🗸 |
| Treatment group | 🗸 | 🗸 | 🗸 | 🗸 | 🗸 |
| Total gms of cannabis in preceding 28 days | 🗸 | 🗸 | 🗸 | 🗸 | 🗸 |
| Cannabis Problems Questionnaire | 🗸 | 🗸 | 🗸 | 🗸 | 🗸 |
| Self-coping and efficacy for quitting questionnaire | 🗸 | 🗸 | 🗸 | 🗸 | 🗸 |
| SF-36 Pain Scores | 🗸 | 🗸 | 🗸 | 🗸 | 🗸 |
| DASS total score | 🗸 | 🗸 | 🗸 | 🗸 | 🗸 |
| Insomnia Severity Index | 🗸 | 🗸 | 🗸 | 🗸 | 🗸 |
| Average Dose of Medication |  |  |  | 🗸 | 🗸 |
| Number of Counselling Sessions |  |  |  | 🗸 | 🗸 |
| log(Weeks Enrolled in Study)^a^ |  | 🗸 |  |  |  |
| **Events Per Variable** | 14.2 | 12.8 | 14.2 | 11.6 | 11.6 |

***a****: offset variable, included to control for exposure*

**Sensitivity Analysis**

The following tables (eTables 3 – 6b) constitute a sensitivity analysis, presenting the results of equivalent Bayesian and NHST analyses side by side for comparison for each outcome variable discussed in the main paper.

**Correlates of duration of treatment**

For this analysis we followed the guidelines described in Chapter 12 of *Applied Longitudinal Data Analysis: Modeling Change and Event Occurrence* by Singer and Willett. Specifically, we conducted discrete-time hazard models, one within a classical Null Hypothesis Significance Testing framework and one within a Bayesian framework, of whether each of the selected correlates of cannabis use disorder were associated with whether and when participants in the trial dropped out of treatment. This analysis amounts to a logistic regression with each discrete time period as its own covariate in the model as well as the nine substantive covariates we are interested in:

1. duration of regular cannabis use (in decades)
2. gender, changed from three-level categorical (male [*n*=97], female [*n*=30], and non-binary [*n*=1]) to binary (male [*n*=97] vs non-male [*n*=31]) for purposes of analysis, due to low numbers in the non-binary group.
3. treatment group (placebo vs nabiximols)
4. average quantity of cannabis used on days when cannabis was used (in grams)
5. score on the cannabis problems questionnaire (z-score)
6. score on the self-coping and efficacy for quitting questionnaire (z-score)
7. score on the SF-36 quality of life scale (z-score)
8. total score on the DASS-21 mood questionnaire (z-score)
9. score on the insomnia severity index (z-score).

These models were level-means coded, such that coefficients for hazard of treatment dropout are reported for each level of categorical predictors rather than representing a comparison of difference in hazard between that level and a reference level (as is the case with the more-common reference-level coding). It is important to note that a term for week 12 was not included in the model, because we deemed any participant who stayed in treatment to week 12 to have completed the trial. This meant that the hazard of treatment dropout in week 12 was zero, which, when the week 12 covariate was included in the model, caused perfect separation, manifesting as and implausibly large hazard estimates and standard errors for the week 12 covariate. In the model reported here and in the manuscript only the weeks 1-11 covariates were included. Another point worth noting is that, based on the guidance in Singer and Willett Chapter 12, we used complementary Log-Log link function for this discrete-time hazard model. This link function is recommended for discrete-time hazard models where the underlying variable is continuous but the actual measurement is discrete. This is the case in our study: participants could drop out of the study on any day of the 84-day study period but the information reported to us about when they dropped out was restricted to weekly windows of time. For the Bayesian analysis the priors used were the defaults for the brms() package in R, that is noninformative ‘uniform(-∞, ∞)’ priors on all intercepts (there are no b coefficients in means-level coded models, only intercepts).

**eTable 3:** Correlates of Duration of Treatment

|  | **Duration of treatment** | |  |
| --- | --- | --- | --- |
| **Covariate Name** | **NHST Analysis**  HR^a^ (95% CI) | **Bayesian Analysis**  HR^a^ (95% CI) | |
| **Time Coefficients**  Week 1^b^  Week 2^b^  Week 3^b^  Week 4^b^  Week 5^b^  Week 6^b^  Week 7^b^  Week 8^b^  Week 9^b^  Week 10^b^  Week 11^b^  **Duration of Regular Use**, in decades | **0.24 (0.10, 0.52)**  **0.26 (0.11, 0.57)**  **0.37 (0.17, 0.75)**  **0.23 (0.08, 0.54)**  **0.25 (0.08, 0.58)**  **0.27 (0.09, 0.64)**  **0.23 (0.07, 0.58)**  **0.37 (0.14, 0.83)**  **0.41 (0.15, 0.93)**  **0.30 (0.09, 0.76)**  **0.32 (0.09, 0.82)**  **0.55 (0.41, 0.72)** | **0.23 (0.09, 0.51)**  **0.25 (0.10, 0.55)**  **0.36 (0.15, 0.73)**  **0.20 (0.07, 0.52)**  **0.22 (0.07, 0.54)**  **0.24 (0.08, 0.62)**  **0.20 (0.06, 0.54)**  **0.34 (0.13, 0.80)**  **0.37 (0.14, 0.87)**  **0.26 (0.08, 0.71)**  **0.28 (0.08, 0.79)**  **0.54 (0.41, 0.71)** | |
| **Gender**, binary, reference group = male (vs non-male^c^) | 0.94 (0.48, 1.77) | 0.90 (0.45, 1.69) | |
| **Treatment group**, binary; reference group = Placebo (vs Nabiximols) | **0.62 (0.38, 0.99)** | **0.60 (0.37, 0.96)** | |
| **Average quantity of cannabis used**, in grams per day | **0.84 (0.71, 0.96)** | **0.83 (0.70, 0.95)** | |
| **Cannabis Problems Questionnaire**, as *z*-score | 0.99 (0.70, 1.40) | 0.99 (0.70, 1.42) | |
| **Self-coping and efficacy for quitting questionnaire**, as *z*-score | 0.96 (0.73, 1.26) | 0.96 (0.73, 1.26) | |
| **SF-36 Pain Scores**, as *z*-score | 1.20 (0.94, 1.54) | 1.21 (0.95, 1.56) | |
| **DASS total score**, as *z*-score | 1.10 (0.78, 1.56) | 1.10 (0.77, 1.55) | |
| **Insomnia Severity Index**, as *z*-score | 1.06 (0.82, 1.40) | 1.07 (0.83, 1.40) | |

***a****: HR = Hazard Ratio: difference in rate of dropout from study given 1-unit change in covariate (when significant 95% CI excludes 1).* ***b:*** *In discrete-time hazard models each discrete time period has its own term in the model, with coefficients expressing the estimated hazard in that time period. If the CI for that time period excludes 1 then there is a non-zero hazard of treatment dropout.* ***c:*** *non-male = female (n=30) + non-binary (n=1), collapsed together due to low numbers in non-binary group*

**Interpretation:** All of the coefficients for each time period had hazard ratios and intervals whose credible intervals excluded 1 (i.e. non-zero hazard of treatment dropout). The longer people had been using cannabis the less likely they were to drop out of treatment early, with every extra decade of regular use associated with an 85% reduction in hazard of dropout (HR=0.54, CI: 0.41, 0.71). The estimated hazard of participants allocated to the nabiximols group dropping out of treatment early was 67% less than in the placebo group (HR=0.60, CI: 0.37, 0.96). Surprisingly the more grams of cannabis per day participants smoked prior to enrolling in the study the LESS their estimated hazard of dropping out early, with each additional gram of cannabis smoked per day associated with an estimated 20% lower hazard of treatment dropout (HR=0.83, CI: 0.70, 0.95).

**Correlates of number of counselling sessions**

As the outcome number of counselling sessions was a bounded count (number of sessions attended out of a maximum possible six), the model we chose for this analysis was a binomial regression, with number of sessions attended by each participant regressed on the nine covariates listed above. To control for exposure (i.e. the length of time participants were able to take part in counseling sessions), the natural logarithm of the number of weeks participants were enrolled in the study was included in the model as an offset variable along with the nine covariates, making ten covariates in total in this analysis. For the prior for the Bayesian analysis we used the default priors supplied by the brms package, that is, extremely broad, weakly-regularising ‘t(3, 0, 2.5)’ for the intercept and b’s.

**eTable 4:** Correlates of number of counselling session attended

|  | **Number of counselling sessions** | |  |
| --- | --- | --- | --- |
| **Covariate name** | **NHST Analysis**  OR^a^ (95% CI) | **Bayesian Analysis**  OR^a^ (95% CI) | |
| **Duration of regular use**, in decades | 1.17 (0.97, 1.40) | 1.17 (0.97, 1.40) | |
| **Gender**, binary, reference group = male (vs non-male^b^) | **1.54 (1.01, 2.33)** | **1.54 (1.01, 2.36)** | |
| **Treatment group**, binary; reference group = Placebo (vs Nabiximols) | **1.41 (1.01, 1.99)** | **1.41 (1.02, 1.97)** | |
| **Average quantity of cannabis used**, in grams per day | **1.10 (1.01, 1.20)** | **1.11 (1.01, 1.21)** | |
| **Cannabis Problems Questionnaire**, as *z*-score | 0.91 (0.73, 1.14) | 0.90 (0.72, 1.13) | |
| **Self-coping and efficacy for quitting questionnaire**, as *z*-score | 0.98 (0.83, 1.17) | 0.98 (0.83, 1.16) | |
| **SF-36 Pain Scores**, as *z*-score | 0.93 (0.78, 1.12) | 0.93 (0.78, 1.12) | |
| **DASS total score**, as *z*-score | 1.05 (0.83, 1.33) | 1.06 (0.83, 1.34) | |
| **Insomnia Severity Index**, as *z*-score | 0.96 (0.79, 1.17) | 0.96 (0.79, 1.17) | |

*Offset variable* *weeks in study not reported.* ***a:*** *OR* ***=*** *Odds Ratio: difference in the odds of attending each extra counselling session given 1-unit change in covariate (when significant 95% CI excludes 1)* ***b:*** *non-male = female (n=30) + non-binary (n=1), collapsed together due to low numbers in non-binary group*

**Interpretation:** The odds of female or non-binary participants attending any given counseling session were 54% (female + non-binary) higher than for male participants (OR=1.54; CI: 1.01, 2.36). The estimated odds of participants allocated to the nabiximols group attending any given counselling sessions were 41% greater than in the placebo group (OR=1.41, CI: 1.02, 1.97). Finally, the more grams of cannabis per day participants smoked prior to enrolling in the study the greater their estimated odds of hazard of attending any given counselling session, with each additional gram of cannabis smoked per day associated with an estimated 11% lower increase in of odds of attending (OR=1.11, CI: 1.01, 1.21).

**Correlates of average dose per day during weeks 2-12 of the trial**

Standard Gaussian regression was used for this analysis, with participants’ average number of sprays of medication (nabiximols or placebo depending on randomisation) during weeks 2-12 of the trial (continuous numeric variable) regressed on the nine covariates. The Bayesian analysis used the default priors supplied by the brms package, that is, extremely broad, weakly-regularising priors: ‘t(3,17.3,10.7)’ for the intercepts and b coefficients, and ‘’t(3,0,10.7)’ for the noise distribution.

**eTable 5:** Correlates of average dose during weeks 2-12 of trial

|  | **Average dose** | |  |
| --- | --- | --- | --- |
| **Covariate name** | **NHST Analysis**  Estimate^a^ (95% CI) | **Bayesian Analysis**  Estimate^a^ (95% CI) | |
| **Duration of Regular Use**, in decades | 0.66 (-1.31, 2.63) | 0.65 (-1.34, 2.72) | |
| **Gender**, binary, reference group = male (vs non-male^b^) | 0.48 (-4.16, 5.12) | 0.44 (-4.15, 5.02) | |
| **Treatment group**, binary; reference group = Placebo (vs Nabiximols) | -0.51 (-4.16, 3.14) | -0.48 (-4.15, 3.22) | |
| **Average quantity of cannabis used**, in grams per day | **1.06 (0.16, 1.96)** | **1.05 (0.16, 1.98)** | |
| **Cannabis Problems Questionnaire**, as *z*-score | -0.66 (-3.06, 1.73) | -0.67 (-2.98, 1.80) | |
| **Self-coping and efficacy for quitting questionnaire**, as *z*-score | -0.78 (-2.63, 1.07) | -0.78 (-2.54, 1.06) | |
| **SF-36 Pain Scores**, as *z*-score | **-2.89 (-4.84, -0.95)** | **-2.89 (-4.74, -0.99)** | |
| **DASS total score**, as *z*-score | -0.77 (-3.28, 1.73) | -0.78 (-3.31, 1.74) | |
| **Insomnia Severity Index**, as *z*-score | 0.54 (-1.55, 2.64) | 0.56 (-1.56, 2.67) | |

***a:*** *estimate is average number of sprays per day during weeks 2-12 of the trial* ***b:*** *non-male = female (n=30) + non-binary (n=1), collapsed together due to low numbers in non-binary group*

An increase of one gram used per day before baseline was associated with an increase in average dose during study weeks 2-12 of 1.05 sprays per day (CI: 0.16, 1.98). An increase of one standard deviation in SF-36 pain factor score (indicating less severe pain) at baseline was associated with an estimated 2.89 fewer sprays per day on average during the trial (CI: -4.74, -0.99). Another way of saying this is that more severe pain at baseline was associated with significantly greater dose of medication.

**Correlates of total abstinence from illicit cannabis in final four weeks or trial and/or of reducing illicit cannabis by 50% or more relative to baseline**

Bernoulli logistic regressions were used for both of these binary outcomes: having abstained entirely from illicit cannabis use during weeks 8-12 of the trial vs not having abstained, and having reduced the frequency of illicit cannabis use (measured in number of days used in the previous 28-day period). These two outcomes were each regressed on the nine predictors mentioned above along with rate of counselling attendance (average number of sessions per fortnight, continuous numeric variable) and average dose during weeks 2-12 of the trial (average sprays per day, continuous numeric variable. For the Bayesian analysis the priors were the default extremely broad weakly-regularising priors created by the brms package for logistic (or Bernoulli) regression: ‘t(3,0,2.5)’ on the intercepts and ‘uniform(-∞, ∞)’ b coefficients.

***Total Abstinence***

**eTable 6a:** Correlates of achieving total abstinence^a^ from illicit cannabis during weeks 8-12 of study

|  | **Abstinence during weeks 8-12** | |  |
| --- | --- | --- | --- |
| **Covariate Name** | **NHST Analysis**  OR^b^ (95% CI) | **Bayesian Analysis**  OR^b^ (95% CI) | |
| **Duration of Regular Use**, in decades | **2.43 (1.22, 5.51)** | **3.03 (1.36, 7.29)** | |
| **Gender**, binary, reference group = male (vs non-male^c^) | 3.51 (0.78, 16.67) | 4.12 (0.75, 24.30) | |
| **Treatment group**, binary; reference group = Placebo (vs Nabiximols) | 2.00 (0.50, 8.66) | 2.27 (0.50, 11.51) | |
| **Average quantity of cannabis used**, in grams per day | 0.97 (0.58, 1.40) | 0.91 (0.53, 1.42) | |
| **Cannabis Problems Questionnaire**, as *z*-score | 0.74 (0.30, 1.82) | 0.72 (0.26, 1.97) | |
| **Self-coping and efficacy for quitting questionnaire**, as *z*-score | 1.37 (0.73, 2.71) | 1.50 (0.75, 3.15) | |
| **SF-36 Pain score**, as *z*-score | 0.47 (0.19, 1.05) | 0.41 (0.16, 1-01) | |
| **DASS total score**, as *z*-score | 0.99 (0.37, 2.66) | 0.98 (0.34, 2.79) | |
| **Insomnia Severity Index**, as *z*-score | 0.73 (0.30, 1.65) | 0.68 (0.26, 1.69) | |
| **Rate of Counseling Attendance**, average number of sessions per fortnight (14 days), M (SD) | **3.79 (1.26, 13.60)** | **5.31 (1.45, 22.19)** | |
| **Average Dose weeks 2-12**, sprays per day, M (SD) | 0.93 (0.85, 1.01) | 0.92 (0.83, 1.01) | |

***a:*** *For the abstinence criterion there were two potential ways of meeting criteria for non-success: (i) using illicit cannabis at least once in the previous 28 days (ii) dropping out of the study early; that is, failing to complete the week 12 research interview.* ***b.*** *OR = Odds ratio. Each coefficient represents the increase in odds of either abstinence or ≥50% reduction associated with a 1-unit increase in the covariate in question. z-score = (raw score – mean)/sd for variable in question.* ***c:*** *non-male = female (n=30) + non-binary (n=1), collapsed together due to low numbers in non-binary group*

**Interpretation:** An increase of one decade of at least weekly cannabis use was associated with an estimated 3.03-fold increase in odds of achieving abstinence in weeks 9-12 (OR=3.03, CI: 1.36, 7.29) and an increase of 1 counseling session per fortnight across the trial was associated with an estimated 5.31-fold increase in odds of abstinence in weeks 9-12 (OR=5.31, CI: 1.45, 22.19).

**≥ 50% reduction in frequency of use relative to baseline**

**eTable 6b:** Correlates of achieving a 50% or greater reduction in frequency of illicit cannabis during weeks 8-12 of study relative to baseline^a^

|  | **Abstinence during weeks 8-12** | |  |
| --- | --- | --- | --- |
| **Covariate Name** | **NHST Analysis**  OR^b^ (95% CI) | **Bayesian Analysis**  OR^b^ (95% CI) | |
| **Duration of Regular Use**, in decades | **2.44 (1.27, 5.28)** | **2.87 (1.39, 6.60)** | |
| **Gender**, binary, reference group = male (vs non-male^c^) | 1.95 (0.51, 7.49) | 2.10 (0.49, 9.46) | |
| **Treatment group**, binary; reference group = Placebo (vs Nabiximols) | **3.29 (1.08, 11.07)** | **4.06 (1.21, 14.95)** | |
| **Average quantity of cannabis used**, in grams per day | 0.81 (0.50, 1.15) | 0.75 (0.46, 1.12) | |
| **Cannabis Problems Questionnaire**, as *z*-score | 0.96 (0.47, 1.94) | 0.96 (0.44, 2.09) | |
| **Self-coping and efficacy for quitting questionnaire**, as *z*-score | 0.87 (0.49, 1.50) | 0.85 (0.47, 1.53) | |
| **SF-36 Pain Scores**, as *z*-score | **0.45 (0.21, 0.88)** | **0.40 (0.18, 0.83)** | |
| **DASS total score**, as *z*-score | 0.69 (0.30, 1.52) | 0.65 (0.27, 1.50) | |
| **Insomnia Severity Index**, as *z*-score | **0.45 (0.22, 0.89)** | **0.39 (0.18, 0.82)** | |
| **Rate of Counseling Attendance**, average number of sessions per fortnight (14 days), M (SD) | **3.04 (1.18, 8.77)** | **3.82 (1.30, 12.15)** | |
| **Average Dose weeks 2-12**, sprays per day, M (SD) | **0.92 (0.86, 0.99)** | **0.91 (0.84, 0.98)** | |

***a:*** *For the* *≥ 50% reduction criterion there were two potential ways of meeting criteria for non-success: (i) reducing number of days of cannabis use in the previous 28 by less than 50% from baseline to week 12, (ii) dropping out of the study early; that is, failing to complete the week 12 research interview.* ***b.*** *OR = Odds ratio. Each coefficient represents the increase in odds of either abstinence or ≥50% reduction associated with a 1-unit increase in the covariate in question. z-score = (raw score – mean)/sd for variable in question.* ***c:*** *non-male = female (n=30) + non-binary (n=1), collapsed together due to low numbers in non-binary group*

**Interpretation:** An increase of one decade of at least weekly cannabis use was associated with an estimated 2.87-fold increase in odds of reducing frequency of cannabis use by 50% or more relative to baseline (OR=2.87, CI: 1.39, 6.60) and an increase of 1 counseling session per fortnight across the trial was associated with an estimated 3.82-fold increase in odds of abstinence in weeks 9-12 (OR=3.82, CI: 1.30, 12.15). Receiving nabiximols was associated with a 4.06-fold increase in the odds of reducing by ≥50% (OR=4.06, CI: 1.21, 14.95) compared to placebo. Less severe pain (increase of one SD) was associated with a 2.5-fold reduction in odds of reducing cannabis use (OR=0.40, CI: 0.18, 0.83). In other words more severe pain was associated with significantly increased odds of reducing illicit cannabis use. More severe sleep problems (higher ISI score of one SD) at baseline were associated with a 2.56-fold decrease in the odds of reducing illicit cannabis use (OR=0.39, CI: 0.18, 0.82). An average increase of 1 spray of medication per day during weeks 1-12 was associated with a 10% reduction in odds of reducing cannabis use by ≥50% (OR=0.91, CI: 0.84, 0.98).

**Priors for Bayesian Analysis**

The priors used for each Bayesian analysis were the default non-informative priors supplied by the brms() package. Details for each are included in eTable 7 below.

eTable 7: Priors used for each Bayesian Analysis

| **Outcome** | **Analysis** | **Priors** |
| --- | --- | --- |
| Duration in treatment, in weeks | Discrete-time hazard model: level-means coded logistic regression with complementary log-log link function | noninformative ‘uniform (-∞, ∞)’ priors on all intercepts (there are no b coefficients in means-level coded models, only intercepts). |
| Number of counselling sessions (out of a maximum of six) | Aggregated binomial regression with natural log of number of weeks client was in study included as an offset | extremely broad, weakly-regularising ‘t(3, 0, 2.5)’ for the intercept and b’s. |
| Average dose during weeks 2-12, in sprays per day | Standard Gaussian multiple regression | extremely broad, weakly-regularising priors: ‘t(3,17.3,10.7)’ for the intercept and b coefficients, and ‘’t(3,0,10.7)’ for the noise distribution. |
| Total abstinence from cannabis during weeks 9-12 of the trial | Bernoulli logistic regression | ‘t(3,0,2.5)’ on the intercepts and ‘uniform(-∞, ∞)’ for the b coefficients |
| ≥ 50% reduction in frequency of cannabis use in weeks 9-12 relative to baseline | Bernoulli logistic regression | ‘t(3,0,2.5)’ on the intercepts and ‘uniform(-∞, ∞)’ for the b coefficients |
